# Acellular approaches for regenerative medicine: on the verge of clinical trials with extracellular membrane vesicles?

**DOI:** 10.1186/s13287-015-0232-9

**Published:** 2015-12-02

**Authors:** Almudena Fuster-Matanzo, Florian Gessler, Tommaso Leonardi, Nunzio Iraci, Stefano Pluchino

**Affiliations:** Department of Clinical Neurosciences; Wellcome Trust-Medical Research Council Stem Cell Institute, University of Cambridge, Clifford Allbutt Building—Cambridge Biosciences Campus, Hills Road, Cambridge, CB2 0PY UK; The EMBL-European Bioinformatics Institute, Wellcome Trust Genome Campus, Hinxton, Cambridge, CB10 1SD UK

## Abstract

Extracellular vesicles (EVs) are a heterogeneous population of naturally occurring secreted small vesicles, with distinct biophysical properties and different functions both in physiology and under pathological conditions. In recent years, a number of studies have demonstrated that EVs might hold remarkable potential in regenerative medicine by acting as therapeutically promising nanodrugs. Understanding their final impact on the biology of specific target cells as well as clarification of their overall therapeutic impact remains a matter of intense debate. Here we review the key principles of EVs in physiological and pathological conditions with a specific highlight on the most recently described mechanisms regulating some of the EV-mediated effects. First, we describe the current debates and the upcoming research on EVs as potential novel therapeutics in regenerative medicine, either as unmodified agents or as functionalized small carriers for targeted drug delivery. Moreover, we address a number of safety aspects and regulatory limitations related to the novel nature of EV-mediated therapeutic applications. Despite the emerging possibilities of EV treatments, these issues need to be overcome in order to allow their safe and successful application in future explorative clinical studies.

## Introduction

Extracellular vesicles (EVs) are lipid membrane vesicles containing a heterogeneous range of molecules. Among those so far described are various classes of nucleic acids as well as soluble and transmembrane proteins [1–3], which are involved in intercellular communication, immune modulation, senescence, proliferation and differentiation among various processes [1–4]. Cells release different types of naturally occurring EVs including exosomes, microvesicles (i.e. shedding vesicles) and apoptotic bodies [5]. The release of EVs is an extremely common and widespread biological process, which is conserved across eukaryotes, bacteria and archaea and is believed to exist in most forms of life [6]. While missing in the past, the field has more recently been using a terminology for EV nomenclature following the mechanisms of vesicle generation [7, 8].

Exosomes originate in multivesicular bodies (MVB). When MVB fuse with the plasma membrane, the intraluminal vesicles are released from the cell and are subsequently referred to as exosomes. Exosomes are reported to be between 40 and 150 nm in size. Microvesicles are shed directly from the plasma membrane and can be larger than exosomes (50–1000 nm) [9]. Apoptotic bodies originate at the cell membrane as cells undergo apoptosis. EVs can interact with target cells using different mechanisms: transmembrane proteins on EVs interact with receptors on the target cell membrane and initiate distinct signalling cascades [10, 11]; or EVs directly fuse with their target cells by (prior to or after) endocytosis/transcytosis, with subsequent release of its content into the cytosol of the target cell [10].

EVs/exosomes have been implicated in a broad, and still largely uncharacterized, range of physiological functions, such as protein clearance [12], immunity [4], signalling [11] and even gene regulation [13], but they have also been identified as important players in pathological processes. EVs/exosomes are thus implicated in infections [14] and cancer [15], and seem also to play a major—yet to be fully characterized—role in neurodegenerative diseases, such as Parkinson’s disease, Alzheimer’s disease (AD), multiple sclerosis (MS), lysosomal storage disorders [16], amyotrophic lateral sclerosis, stroke and prion disease [17]. Implication in such a high number of both pathological and physiological functions makes EVs not only potential biomarkers of diseases but also good candidates for the development of new cell-free (acellular) therapies.

## EVs and regenerative medicine

Regenerative medicine aims at the restoration of a damaged or malfunctioning tissue by applying cell-based or stem cell-based therapies, small molecules and tissue engineering-based or material-based approaches [2]. Recent research focuses on strategies that allow functional restoration of a damaged tissue by cell-free (acellular) approaches or using autologous cell and tissue sources [2] (UKRMP Hub for Acellular (smart material) approaches for therapeutic delivery; http://www.ukrmp.org.uk/hubs/acellular/acellular-hub-news-and-events/). At the same time, latest developments in the field of EVs have uncovered novel functions for EVs in various processes including angiogenesis, extracellular matrix (ECM) remodelling and regulation of immune responses [11, 18, 19], which may also be of interest for tissue engineering [2]. Taken together, EVs derived from various cell types are thought to play an important role in regeneration of various disease models. Although we are far from effective therapies and only a few clinical trials have been started in most cases, it is worth discussing promising results obtained in some relevant animal disease models.

### Myocardial infarction

Myocardial infarction leads to diffuse death cardiomyocytes [20], which are replaced by a collagen-based scar due to the negligible regenerative capacity of the adult mammalian heart. Necrosis of ischemic cardiomyocytes also triggers an intense inflammatory reaction that serves to clear the wound from dead cells and matrix debris and contributes to formation of a collagen-based scar [21].

**Fig. 1:**
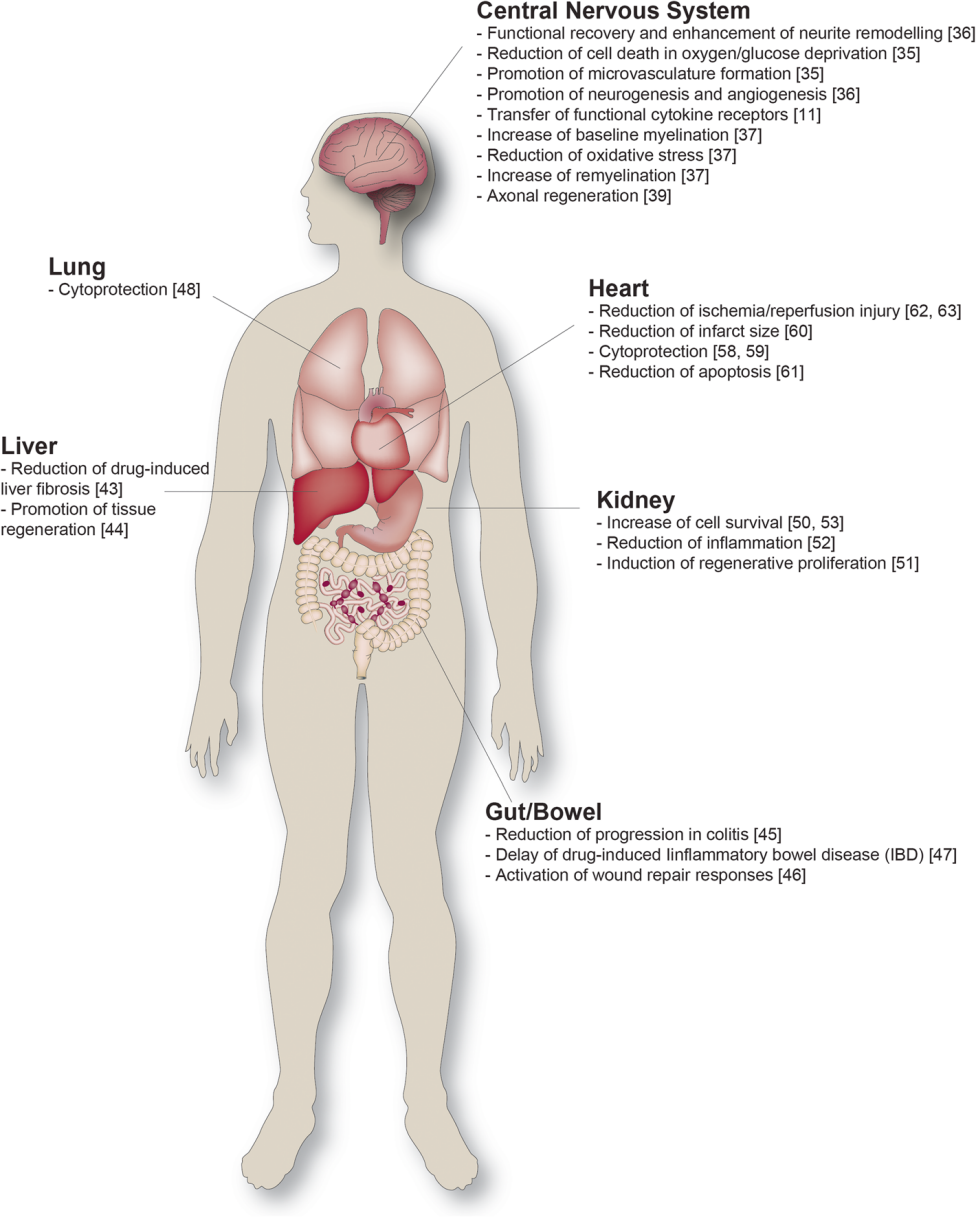
Overview of effects of EV therapeutics in animal disease/injury models. Data in the figure include evidence from EVs collected from DCs, MSCs and neural stem cells

Indirect evidence suggests that EVs participate in the processes of cardiovascular diseases from atherosclerosis and myocardial infarction to heart failure. Consequently, they are worth exploiting for therapy, for prognosis and as biomarkers for health and disease [22]. Several experimental data support this concept. As such, mesenchymal stem cell (MSC)-derived EVs have been demonstrated to improve recovery when injected into laboratory animals with experimental myocardial infarction and to reduce the infarct size area by promoting neoangiogenesis [23]. Besides, EVs have been confirmed as the cardioprotective component in the MSC secretome [23]. Similar results have further highlighted the importance of EVs not only as pro-angiogenic cargo particles but also as protector factors from senescence and cell death [24]. Furthermore, intracardial injections of conditioned medium from MSC overexpressing the survival gene (Akt1Akt-MSC) limited infarct size and improved ventricular function by reducing the rate of apoptosis [25, 26]. MSC-derived EVs displayed the same effects in mice following myocardial ischemia/reperfusion injury by activating the PI3K/Akt pathway, and in turn increasing ATP levels and reducing oxidative stress [23, 27] (Fig. 1).

### Acute kidney injury

Acute kidney injury (AKI) is a syndrome characterized by the acute loss of kidney function that leads to increased serum creatinine or oliguria. To mimic the different clinical settings of AKI and to set up and/or improve possible new treatments, several experimental animal models have been developed in which EVs have been tested as a new experimental therapeutic option [28]. Human MSC-derived EVs have been reported to stimulate proliferation and apoptosis resistance of tubular epithelial cells in vitro [29]. In vivo, morphological and functional recovery of different experimental animal models of acute and chronic kidney is observed after injection of MSC-derived EVs, in a manner comparable with that observed after transplantation of parental MSCs. Interestingly, the pretreatment of MSC-derived EVs with RNase (i.e. to inactivate their RNA cargoes) abrogated these protective effects. Kidney regeneration has been also observed in an EV-xenotransplantation study [29, 30]. Further studies confirmed the protective effects of EVs in kidney injury models by a CX3CL1-mediated mechanism [31–33], by inhibition of apoptosis through the regulation of extracellular signal-regulated kinase (ERK) 1, ERK 2 and mitogen-activated protein kinase (MAPK) pathways [33], or by transferring anti-apoptotic microRNAs (miRNAs) [34]. Moreover, injected EVs are able to produce an increase in proliferation, as reported in an AKI model [35] (Fig. 1).

### Neurological disorders

Extensive studies have implicated EVs in a broad range of neurological disorders, and in some cases their potential value as targets for treatment development and as markers for diagnosis. For example, in a model of MS, Pusic et al. [36] demonstrated that dendritic cell (DC)-derived EVs bear cytoprotective effects, as they promote remyelination of damaged nerve fibres. Moreover, Schwann cell-derived EVs mediated protective effects and induced axonal regeneration in in-vitro and in-vivo models of sciatic nerve injury [37]. The relevance of EVs as mediators for intercellular communication in the peripheral nervous system between Schwann cells and axons and its importance in axonal maintenance and regeneration after nerve damage is well described [38]. This EV-mediated communication also exists in the central nervous system (CNS), where oligodendrocyte-derived exosomes contribute to the neuronal integrity by releasing neurotransmitters [39] and embody a signalling moiety involved in glia-mediated trophic support to axons [40]. In the CNS, this oligodendrocyte–neuron communication mediated by EVs has also been demonstrated to promote myelination as described recently by Pusic and Kraig [41], who have attributed part of this effect to exosomes containing miR-219.

Some advances have also been made in the field of AD. Continuous administration of exosomes derived from wild-type neuroblastoma or primary neurons in the hippocampus ameliorates amyloid-beta (Aβ) pathology and synaptic dysfunction in APP_SweInd_ mice. The beneficial action of exosomes is associated with a marked decrease in Aβ burden as well as with a significant synaptophysin immunoreactivity rescue in AD mice. Neuroprotection has been ascribed to the capability of exosomes to trap Aβ and to promote its clearance by microglia [42]. Finally, a protective effect in AD has also been reported for MSC-derived EVs, since they carry an active version of neprelysin, one of the key Aβ-degrading enzymes in the brain. Some experiments conducted in N2A cells overexpressing Aβ demonstrated that after EV treatment both extracellular and intracellular levels of Aβ were reduced [43]. Some improvements mediated by EVs have also been described in stroke pathology. A recent study in a middle cerebral artery occlusion (MCAO) rat stroke model reported the possibility that MSCs might communicate with brain parenchymal cells via exosome-mediated miR-133b transfer, leading to specific gene expression (i.e. connective tissue growth factor) regulation that in turn enhanced neurite outgrowth and contributed to functional recovery [44] (Fig. 1).

### Gastrointestinal diseases

Protective effects involving regeneration and/or regulation of immunity are some of the functions that EVs seem to mediate in gastrointestinal diseases. The application of MSC-derived EVs resulted in decreased liver damage in mouse models of drug-induced damage [45, 46]. In a model of experimentally induced colitis, the injection of EVs derived from gut microbiota regulated intestinal immunity and haemostasis [47]. Furthermore, administration of an exogenous glucocorticoid-regulated protein annexin A1 (ANXA1) mimicking peptide encapsulated within targeted polymeric nanoparticles significantly accelerated healing of mucosal wounds in experimentally DC-derived induced colitis [48]. In another approach, transforming growth factor (TGF) beta1 gene-modified exosomes delayed drug-induced inflammatory bowel disease [49] (Fig. 1).

### Graft rejection

Immune response constitutes a major issue in the context of cell therapies and tissue engineering. Several cells are involved including T cells, macrophages and DCs, each with different functions including phagocytosis, cytokine production and antigen presentation. EVs have been shown to modulate innate immune response, turning them into good candidates to prevent rejection of a graft [50]. On the other hand, MSC-derived exosomes are able to induce a shift in macrophages toward an anti-inflammatory M2 phenotype [51] and to directly postpone allograft rejection in a rat kidney transplantation model [52]. Finally, the importance of exosome-mediated signalling in the immunological haemostasis of the CNS is highlighted by evidence of a transfer of oligodendrocyte-derived exosomes to microglia occurring differentially depending on the immunological profile of microglia [53] (Fig. 1).

## EVs as drug delivery tool

In addition to the use of EVs as natural modifiers of disease, recent literature also describes the use of EVs as (naturally occurring) non-synthetic drug delivery systems, due to their inherent lower immunogenicity and toxicity as well as their intrinsic homing and loading abilities [1, 5]. Taking advantage of these properties, EVs loaded by electroporation have been demonstrated to functionally transfer small interfering RNAs (siRNAs) and/or miRNAs to target cells [54–57]. In addition to artificial loading mechanisms, the endogenous cellular machinery responsible for secreting miRNAs into EVs can be exploited in order to load therapeutically relevant siRNA and miRNA in EVs. Next to the loading, functional delivery into target cells has also been shown by several groups [57–59].

Moreover, other intrinsic properties of EVs—such as their lipid composition, which enhances their stability in circulation [60]; their protein content, which slows EV clearance acting as inhibitors of complement and phagocytosis [61, 62]; and their ability to cross the blood–brain barrier (BBB) [36, 54]—make them ideal vehicles for delivery of exogenous therapeutic molecules ranging from nucleic acids to other bioactive small molecules. In fact, this concept has been already tested by loading EVs with drugs such as doxorubicin in breast cancer xenografts [63].

The delivery of exogenous biomolecules requires a suitable strategy to efficiently load the molecule into EVs. Loading strategies can be divided into ex vivo strategies, where circulating EVs are purified and then loaded with the appropriate cargo, and in vitro strategies, where cargo is incorporated during vesicle biogenesis.

Among the ex vivo strategies, the most broadly applied is electroporation of EVs, a technique used to deliver small-molecule drugs [63–65] and siRNA [54, 55]. However, this technique still requires further optimization, because currently the electroporation conditions may induce siRNA precipitation and yield low siRNA incorporation into EVs [66]. On the other hand, in vivo strategies can be further divided into passive and active loading approaches. The passive loading approaches exploit the endogenous trafficking mechanisms of the cell and loading is achieved by overexpression of the cargo molecule. Although using cell machinery represents a clear potential advantage, this method also presents challenges because undesirable cargoes might also be loaded into EVs, leading to unexpected (off vs*.* toxic) effects in target cells [67]. The active loading approaches are aimed at increasing the concentration of the cargo specifically within the vesicles. The most commonly employed method relies on the creation of a fusion protein between the molecule of interest and a protein that is natively expressed in EVs. One example is the N terminus of lactadherin C1C2 domain, which is localized in the surface of the vesicles and has been fused to different proteins or peptides [54, 68].

Finally, another noteworthy loading method for nucleic acids consists of exploiting viral packaging systems using hybrid vesicles called vexosomes. For example, non-enveloped viruses such as adenoassociated virus (AAV) [69] and hepatitis A virus [70] can be incorporated into EVs during propagation. Vexosomes containing AAV within the EVs can be less immunogenic due to the EV component and, at the same time, the AAV component is effective for gene delivery with long-term stability in non-dividing cells [67]. As an alternative to loading of RNA molecules, the loading of medication for regenerative purposes could be an option. For example, curcumin, a natural anti-inflammatory drug, protects mice from lipopolysaccharide (LPS)-induced brain inflammation and from progression of myelin oligodendrocyte glycoprotein (MOG) peptide-induced experimental autoimmune encephalomyelitis (EAE) upon intranasal administration in the form of curcumin-loaded EVs [71].

When considering EVs as good candidates for therapeutics, one important aspect is their ability to spread throughout the organism and to reach their target organs. EVs have been administered intravenously, subcutaneously, intranasally and systemically in mice. When administered intravenously in laboratory animals, as early as 30 minutes after injection EVs are found in the spleen, liver, lung and kidneys, with some signal detectable in the brain, heart and muscle [72, 73]. Signal is no longer detected in blood after 3 hours. Importantly, intravenously or subcutaneously administered vesicles preferentially bind to distinct cell types. As such, biotinylated B-cell-derived EVs are primarily taken up by hepatic and splenic macrophages 5 minutes after systemic administration, with a rapid elimination of EVs from the circulation, which resulted in a half-life of 2 minutes [74]. Locally administered EVs may achieve very high local concentrations at target sites. In fact, intranasal administration of vesicles offers an interesting example which has already been tested in mice [75]. On the other hand, many other promising routes for administration (e.g. intrathecal, intracerebral or intraventricular) have not yet been tested [67]. In the context of clinical trials and according to general considerations stated by the US Food and Drug Administration (FDA) (http://www.fda.gov/downloads/drugs), depending on the route of administration, acute and repeated dose, local toxicity studies with histological evaluation should be conducted either in one or even two animal species. Route-specific considerations should be also taken into consideration (i.e. intravenous; compatibility with blood should be evaluated).

## Clinical trials

In this novel field the development of scientific research has just begun, which is reflected by the limited number of early-phase clinical trials that have been undertaken over the past two decades in order to establish EVs as therapeutic agents [76–80] (Table 1).

**Table 1:** Summary of current clinical trials with extracellular vesicles and their applications

EV source	Application	Proposed mechanism	Clinical phase	Status	Reference
Dendritic cell-derived exosomes	Metastatic melanoma	Immunisation with autologous exosomes pulsed with MAGE 3	Phase I	Completed showing safety and feasibility	[76]
Ascite-derived exosomes	Colorectal cancer immunotherapy	Immunisation with ascite-derived exosomes and GM-CSF	Phase I	Completed showing safety and feasibility	[77]
Dendritic cell-derived exosomes	Non-small cell lung cancer (NSCLC)	Immunisation with autologous exosomes loaded with MAGE antigens	Phase I	Completed showing safety and feasibility	[80]
MSC-derived exosomes	Graft-versus host disease (GvHD)	Donor-derived exosomes to recapitulate the immunomodulatory properties of MSCs	Individual patient	Symptoms improved and stabilized for several months. Patient died of pneumonia after 7 months	[78]
Dendritic cell-derived exosomes	Large-scale interferon-gamma vaccines	Immunisation with exosomes loaded with tumour antigens	Phase II	Ongoing	[80]

*EV* extracellular vesicle, *GM-CSF* granulocyte–macrophage colony-stimulating factor, *MAGE* melanoma-associated antigen, *MSC* mesenchymal stem cell

These data seem promising for future EV applications, even if none of these studies [76–80] can truly be considered as addressing regenerative medicine. However, the lack of published clinical trials in the context of regenerative medicine does not mirror the scientific and financial interests, as different stem cell companies are undertaking significant efforts to develop EV therapeutics derived from stem cells.

### EVs: from bench to patients

Several issues must be considered and different problems need to be solved before finally translating EVs into clinics.

#### Manufacturing vesicles for therapeutic use

The choice of an appropriate producer cell type must be made. Mammalian vesicles can be either produced by cell lines or by primary cells. Cells are constantly secreting EVs, so producing them requires cell culture, as does the manufacture of other biologics. Nevertheless, unlike that of recombinant biopharmaceuticals, genetic manipulation of producer cells is not required for EV production because all cells secrete them naturally. Of the three companies developing EVs for commercial use nowadays, two are producing them from primary cells that are being explored therapeutically—Capricor Inc., specialized in cell therapy for applications in heart and muscle diseases (http://capricor.com); and ReNeuron Group PLC, specialized in cell therapy for applications neurological and ischemic conditions (http://www.reneuron.com)—and just one company has attempted to start de novo EV therapeutic development—Anosys Inc., started with the aim of manufacturing autologous DC-derived EVs as cancer vaccine (http://chromos.com).

However, it is important to bear in mind the pros and cons of both options: while cell lines are less characterized and may induce oncogenic effects, primary cells have been extensively studied and reduce the risk of immunological rejection [81], which in some cases has been avoided by using autologous EVs [76, 77]. In general, primary cells have lower vesicle yield and limiting passage numbers, which make them harder to use to generate a cell bank. It is also worth mentioning that the US FDA has approved some cell lines for vaccine production. These cells have undergone extensive testing for oncogenic potential and for the presence of endogenous viruses. Particularly, Crucell (now Janssen; http://crucell.com/about-us) has developed a proprietary fully tested PER.C6® human cell-line technology previously used for vaccine production. Some other EVs sources like non-mammalian cells (bacteria, yeast and plant cells) are also considered, but their clinical potential is currently being studied [67].

Isolation techniques represent one of the major issues concerning EV therapeutics. Currently, there is no reliable method for either basic research or for more translational applications [81]. So far, the most common strategies to purify EVs for clinical applications have been ultrafiltration to concentrate the conditioned medium followed by ultracentrifugation into a sucrose cushion [82] or a polyethylene glycol 6000 precipitation method [78]. However, undesirable co-isolation of contaminants (i.e. protein aggregates and incomplete separation of vesicles from lipoproteins) is likely to occur. Overcoming this issue, currently chromatography-based methods appear very promising. Specifically, size exclusion chromatography (SEC) has been demonstrated to be efficient for EV isolation in a single-step process [83, 84].

These or any other methods need to be reproducible, with short processing times, and capable of maintaining EV functional properties and avoiding contaminants and impurities. Depending on the application, aspects such as the purity or the homogeneity/heterogeneity of the sample must be also taken into account, since different isolation techniques have been shown to influence EV integrity and biodistribution in vivo*.* Finally, storage conditions for EVs must be optimized and validated. For example, in order to conserve EV functional and physical properties, isotonic buffers to prevent pH shifts during freezing and thawing procedures and during storage should be used. Storage temperature also has to be established. Besides, EVs can unexpectedly bind to certain materials, so containers for long storage have to be chosen carefully since they can affect the quality of the sample.

#### Characterization and evaluation of quality aspects

The EV content of given samples should be quantified and the average size distribution and their protein concentration have to be determined [81]. Methods for characterization are emerging and developing. Some routinely used methods include transmission electron microscopy (TEM), fluorescence microscopy, flow cytometry or nanoparticle tracking analysis (NTA). Since each method shows its own limitations, it is important to take into consideration the original sample from where EVs will be isolated, because different efficiency rates have been observed for each method depending on the source of the sample [84]. As a general rule, the presence of at least three or more categories of EV-specific marker and non-EV-specific proteins should be analysed in a semi-quantitative manner. Additional markers to identify the presence of impurities should be included.

#### Basic biological and pharmaceutical questions must be covered

A deeper knowledge of the action and biological function of EVs is required. Biological assays are needed to test them for therapeutic applications. Assays must be designed specifically for each application, considering all aspects regarding their interpretation, feasibility and reproducibility. Importantly, dose-finding studies as well as cytotoxicity assays have to be performed. Accordingly, the route of administration also has to be defined. As already discussed, this can affect the biodistribution of the EVs so the administration route has to be carefully analysed for each of the particular applications considered. Immune response and tumorigenic effects also need to be checked in a systematic way.

The need for standardization concerning these first three issues remains a major issue for translational application of EVs. Current companies are putting their efforts towards the development and improvement of an adequate infrastructure (technical equipment according to pharmaceutical manufacturing standards) and a quality management system (implementation of manufacturing procedures according to pharmaceutical standards).

Complicated regulatory issues must also be solved. Current legislation at least in the United States and Europe does not provide specific regulation of EV-based therapies, and thus the definition of ‘biological medicine’ (a medicine that contains one or more active substances made by or derived from a biological cell) is applicable for EV-based therapeutics. This pharmaceutical classification harbours special challenges with regard to pharmaceutical manufacturing and preclinical safety testing. Following standardized production, biological medicinal products have to be characterized by a combined approach of testing the expected active substances (i.e. safety, pharmacology, pharmacodynamics and toxicology testing) and the final medicinal product together with a tight assessment of the pharmaceutical production processes and associated controls. Production has to be performed under compliance with GxP regulations (Good Manufacturing/Good Laboratory/Good Distribution/Good Clinical/Good Scientific Practice or GMP/GLP/GDP/GCP/GSP). In fact, regulatory agencies are monitoring manufacturers through periodical inspections with respect to their adherence to GxP standards such as the FDA’s Center for Biologics Evaluation and Research (CBER) and FDA in the United States, the Competent Authorities of European Member States and the European Medicines Agency (EMA) in Europe, the Ministry of Health, Labour and Welfare (MHLW) in Japan and the Therapeutic Goods Administration (TGA) in Australia.

#### Commercialization

Biotechnology companies are moving their activity towards therapeutic applications for EVs. Several companies have already commercialized methods for isolation and purification (e.g. System Biosciences, Life Technologies, Qiagen, HansaBioMed, Cell Guidance Systems and Exosome Diagnostics) [67]. However, several technical and safety issues must be solved before EVs are finally translated into clinics. Diagnostics is, on the other hand, a very interesting and promising application for EVs that some companies are already exploiting. The majority of these companies are focusing mainly on cancer, since a lot of work has already been done reinforcing the idea of EVs as good biomarkers for diagnosis or to predict or monitor a patient’s response to treatment [85]. A good case is Exosome Diagnostics (http://www.exosomedx.com), which is offering pharma services for clinical trials, from biomarker discovery through validation and companion diagnostics, so far being the only platform that allows exploration and validation of RNA and DNA from biofluids.

Although clinical evaluation of EV therapeutics is still at an early stage, it is rapidly expanding.

## Conclusions

Promising results obtained over the last decades highlight EVs as candidates for therapeutic approaches in regenerative medicine. Preclinical and laboratory data show promising effects of EV-mediated therapy in relevant models of neurological, cardiac and intestinal diseases. Major aspects of traditional regenerative medicine approaches have been demonstrated to be modifiable by cell-free approaches facilitating EVs, including ECM modification, angiogenesis, tissue protection and immunomodulation.

Although some clinical trials have been already conducted to evaluate the impact of EVs in models of cancer, there is a great expectation from the results in the field of regenerative medicine. To further progress in the field of EVs, continuing efforts must be done to overcome all issues raised and discussed in this review, thus allowing EVs to be translated from basic research to clinics, especially in the context of regenerative medicine.

EV-mediated therapy, if able to overcome the limitations named, could combine designed, personalized and specific medicine. EVs display a cell-free approach to regenerative medicine mirroring the results that have been demonstrated for somatic and stem cell approaches.

For future considerations, recent developments in the understanding of the preclinical and academic knowledge of the heterogeneity of EVs underline the need for improved standardizations of the protocols used for isolation and storage, and definition of the criteria for characterization and quality control. The aspects named must be taken into account when considering EVs as candidates for regenerative medicine. Thus, it is important to well define the role that they exert in essential processes important for regeneration and the methods for delivery. These issues are the current matter of discussion and the main concern of experts in the field nowadays.

## Note

This article is part of a thematic series on *Extracellular vesicles and regenerative medicine* edited by Jeffrey Karp, Kelvin Ng and Armand Keating. Other articles in this series can be found at http://stemcellres.com/series/EVRM.

## Abbreviations

AAV: Adenoassociated virus
Aβ: Amyloid beta
AD: Alzheimer’s disease
AKI: Acute kidney injury
BBB: Blood–brain barrier
CNS: Central nervous system
DC: Dendritic cell
EAE: Experimental autoimmune encephalomyelitis
ECM: Extracellular matrix
EMA: European Medicines Agency
ERK: Extracellular signal-regulated kinase
EV: Extracellular vesicle
FDA: Food and Drug Administration
LPS: Lipopolysaccharide
MAPK: Mitogen-activated protein kinase
MCAO: Middle cerebral artery occlusion
MHLW: Ministry of Health, Labour and Welfare
miRNA: MicroRNA
MOG: Myelin oligodendrocyte glycoprotein
MS: Multiple sclerosis
MSC: Mesenchymal stem cell
MVB: Multivesicular bodies
NTA: Nanoparticle tracking analysis
SEC: Size exclusion chromatography
siRNA: Small interfering RNA
TEM: Transmission electron microscopy
TGA: Therapeutic Goods Administration
TGF: Transforming growth factor
